# Role of Mast Cell-Derived Adenosine in Cancer

**DOI:** 10.3390/ijms20102603

**Published:** 2019-05-27

**Authors:** Yaara Gorzalczany, Ronit Sagi-Eisenberg

**Affiliations:** Department of Cell and Developmental Biology, Sackler Faculty of Medicine, Tel Aviv University, Tel Aviv 69978, Israel; gyaara@tauex.tau.ac.il

**Keywords:** adenosine, cancer, mast cells, tumor microenvironment

## Abstract

Accumulating evidence has highlighted the accumulation of mast cells (MCs) in tumors. However, their impact on tumor development remained controversial. Indeed, cumulative data indicate an enigmatic role for MCs in cancer, whereby depending on the circumstances, which still need to be resolved, MCs function to promote or restrict tumor growth. By responding to multiple stimuli MCs release multiple inflammatory mediators, that contribute to the resolution of infection and resistance to envenomation, but also have the potency to promote or inhibit malignancy. Thus, MCs seem to possess the power to define tumor projections. Given this remarkable plasticity of MC responsiveness, there is an urgent need of understanding how MCs are activated in the tumor microenvironment (TME). We have recently reported on the direct activation of MCs upon contact with cancer cells by a mechanism involving an autocrine formation of adenosine and signaling by the A3 adenosine receptor. Here we summarized the evidence on the role of adenosine signaling in cancer, in MC mediated inflammation and in the MC-cancer crosstalk.

## 1. Mast Cells (MC) and Cancer

Though best known for their involvement in allergic disorders, accumulating evidence highlights mast cell (MC) involvement in multiple inflammatory disorders, including cancer [1,2]. MCs infiltrate tumors and constitute an important part of the tumor microenvironment (TME) [3]. However, their impact on tumor development remained controversial. Indeed, cumulative data indicate an enigmatic role for MCs in cancer, whereby depending on the circumstances, which still need to be resolved, MCs function to promote or restrict tumor growth [4,5,6,7,8]. By responding to multiple stimuli MC release multiple inflammatory mediators that have the potency to promote or inhibit malignancy. Hence, by evoking an acute immune response, MCs may contribute to restriction of the tumor. However, by provoking inflammatory responses and releasing angiogenic and growth promoting factors, MCs may enhance cancer cell proliferation and enhance tumor progression. Therefore, MCs seem to possess the power to define tumor projections [9]. Given the remarkable plasticity of MCs, whereby their phenotype changes depending on the local microenvironment, and the repertoire of their released mediators is stimulus-dependent [10,11,12], it is envisioned that depending on the cancer type or TME composition, MC may change their patterns from anti to pro tumorigenic features [2]. In the majority of cases, the numbers of tumor infiltrating MCs correlate with enhanced tumor growth and tumor invasion, increased vascularity and poor prognosis. Such is the case in pancreatic cancer, one of the most lethal cancer types, for which clinical data and supporting studies in animal models and in cell culture have demonstrated that MC infiltration correlates with worse prognosis [13,14,15,16,17,18]. Moreover, attenuated pancreatic tumor growth was documented in MC-deficient mice whereby tumor growth was accelerated upon reconstitution with wild-type bone marrow-derived MCs (BMMCs) [14]. Indeed, in addition to their ability to influence tumor growth through the release of pro-tumorigenic mediators, MCs were also shown to deliver proteins, including their c-kit receptor to the tumor cells by means of extracellular vesicles. Specifically, exosomes derived from the human MC line, HMC-1 are taken up by A549 non-small cell cancer lung epithelial tumor cell line, promoting their proliferation, by transferring kit protein and mRNA via exosomes [19]. However, the underlying mechanisms of MC activation within the TME remain largely unresolved. We have recently demonstrated the involvement of autocrine/paracrine signaling of extracellular adenosine in the MC-cancer crosstalk [20].

## 2. Adenosine

Adenosine is a nucleoside consisting of adenine (the purine base) in glycosidic linkage with the sugar ribose. Serving as a precursor and metabolite of adenine nucleotides, adenosine plays a central role in energy transfer and metabolism in living organisms. Adenosine is also generated and released from cells, specifically by either nucleoside transporters [21] or by microvesicles [22], or non-specifically, following the release of adenine nucleotides during apoptosis or necrosis [23]. Adenosine is then formed through the action of the ecto-nucleoside triphosphate diphosphorylases (E-NTPDases), CD39 and the ecto-5’-nucleotidase, CD73, that convert released ADP/ATP to AMP and AMP to adenosine, respectively [24]. As such, adenosine also acts as an extracellular signal that modulates a wide spectrum of physiological functions [25,26]. Indeed, adenosine affects the central nervous system [27], the immune system [28], endocrine system [29] and cardiovascular system [30]. Adenosine also impacts numerous pathological processes, including cancer [31,32]. During homeostasis, adenosine is present in the extracellular space at low concentrations. Metabolically stressful conditions dramatically increase its extracellular levels, which can rise from low nano-molar to micro-molar concentrations [33]. Adenosine concentrations are also regulated by the enzyme adenosine deaminase that degrades intracellular adenosine to inosine, but it also functions as an ectoenzyme in some cells, such as dendritic cells [34] and lymphocytes [35]. Finally, adenosine concentrations are regulated by adenosine uptake by specific transporters, including the equilibrative nucleoside transporters (ENTs) and the concentrative nucleoside transporters (CNTs) [21].

## 3. Adenosine Receptors

Adenosine functions are mediated by four G-protein coupled receptors (GPCRs), designated A1 adenosine receptor (A1R), A2a adenosine receptor (A2aR), A2b adenosine receptor (A2bR), and the A3 adenosine receptor (A3R) [36]. The receptors display different affinities to adenosine and couple to distinct G proteins [37]. A1 and A3 receptors couple to Gi, Gq, and Go, whereas A2a and A2b receptors are coupled to Gs or Gq. Therefore, the functional outcome of adenosine may change depending on the profile of adenosine receptor expression and the concentration of adenosine, which may change depending on the circumstances. As mentioned above, adenosine concentrations increase under stressful conditions and so do the expression patterns of the adenosine receptors. For example, NF-κB promotes A1R expression following oxidative stress [38], whereas hypoxia and inflammatory factors, such as the pro-inflammatory cytokines; tumour necrosis factor (TNF)-α [39], interferon (IFN)-γ [40], interleukin (IL)-1β [41], and the reactive oxygen species-generating enzyme Nox4 [42], upregulate A2bR. Since A2bR has the lowest adenosine affinity [43], the contribution of this receptor’s signaling is particularly significant during pathological conditions.

## 4. Adenosine and Cancer

Adenosine concentrations in the TME are significantly higher than in normal tissue alluding to the role of extracellular adenosine in controlling tumor progression [31,44,45,46]. In particular, extracellular adenosine and its generating enzymes CD39 and CD73 have been implicated in suppressing anti-tumor immunity and at the same time stimulating angiogenesis [47]. Indeed, cancer cells express CD73 whose expression is upregulated by hypoxia and is further upregulated through the signaling of the so formed extracellular adenosine, thus generating positive feedback [48]. Increased expression of CD39 has also been reported in several tumors, including pancreatic cancer [49]. Adenosine then dampens cytotoxic activities of T lymphocytes and natural killer (NK) cells and limits the capacity of tumor-infiltrating myeloid cells to evoke anti-tumor immune responses [46]. Extracellular adenosine also inhibits phagocytosis, as well as neutrophil degranulation, adhesion to endothelial cells and superoxide production (Reviewed in [45]). Thus, an adenosine-rich TME is an immunosuppressed TME that features strong inhibition of anti-tumor T and NK cells and adenosinergic signaling emerges as an important immunometabolic drug target [50,51]. High expression of CD39 and CD73 has been linked with poorer clinical outcomes in a number of cancer types, including triple negative breast, lung, ovarian, kidney, gastric cancer, and melanoma [52]. Accordingly, both CD39 and CD73 have been marked as a drug target for cancer therapy [47,48]. Adenosine also directly affects tumor cells, though the literature is split between data showing anti tumorigenic effects of adenosine signaling to pro tumorigenic [46], which may reflect the strong dependence of adenosine signaling on the profile of adenosine receptor expression. In particular, the A2bR and the A3R have been implicated in cancer modulation. Divergent effects were reported for the A3R, that has been reported to be upregulated in primary tumors and metastases [53,54]. Activation of the A3R was shown to enhance tumor cell migration and invasion [55] and stimulate melanoma and colorectal cell proliferation [56]. On the other hand, using a different model, A3R activation was shown to inhibit colorectal and melanoma cell proliferation [46]. Finally, A3R activation was shown to induce apoptosis of lung cancer cells [57] and hepatocellular carcinoma [58]. In fact, CF102, a specific agonist of the A3R is under clinical development as an anti-cancer drug [58]. However, in view of our studies showing down-regulation of the A3R in response to prolonged triggering, the anti-tumorigenic activity of CF102 might be due to receptor down-regulation rather than due to activation of this receptor [20]. The function of the A2bR, which is likewise highly expressed in tumors [59], has been primarily linked with stimulation of tumor proliferation, immunosuppression and ability to induce epithelial-mesenchymal transition (EMT) [46,59]. As mentioned above, given the low affinity of this receptor to adenosine, the contribution of the A2bR to the overall signaling of adenosine would rise under conditions of increased concentrations of extracellular adenosine.

## 5. Adenosine and MC Inflammation

Adenosine has long been incriminated in the pathogenesis of bronchoconstriction of MC-dependent allergic asthma. Inhaled adenosine provokes bronchoconstriction in atopic and asthmatic individuals, but not in normal subjects, implicating adenosine in the induction of airway hyperresponsiveness (AHR), a hallmark of asthma [60]. Post-mortem analysis has indicated that the number of degranulated MCs in airway smooth muscle was greatest in persons who died from asthma [61]. Finally, prominent secretion of adenosine evoked within 60 sec with stimulants, was previously observed in MCs [62]. Animal models have substantiated these results demonstrating that adenosine deaminase (ADA)-deficient mice exhibit extensive lung MC degranulation concurrent with elevated adenosine levels [63]. Assessment of airway responses elicited by adenosine revealed robust airway responses in wt mice, but a significantly attenuated response in A3R-deficient, and MC-deficient mice [64]. Furthermore, AHR was developed in MC-deficient mice, which were reconstituted with wt, but not with A3R^−/−^ MCs [65]. However, while the involvement of adenosine in AHR in human is established, and the evidence supports the involvement of the A3R in this process, the identity of the human adenosine receptor(s) responsible for the adenosine-induced bronchospasm is less clear. In in vitro studies, adenosine potentiated the release of histamine from immunologically activated human lung MCs. However, at higher concentrations secretion was inhibited [66,67]. This duality most likely reflects the variable outcome of the distinct contributions of the MC expressed adenosine receptors. For more information, the reader is referred to a recent review that summarizes the available knowledge on adenosine signaling in MC degranulation and its role in asthma [68].

## 6. Adenosine Signaling during Cancer-MC Crosstalk

**Figure 1:**
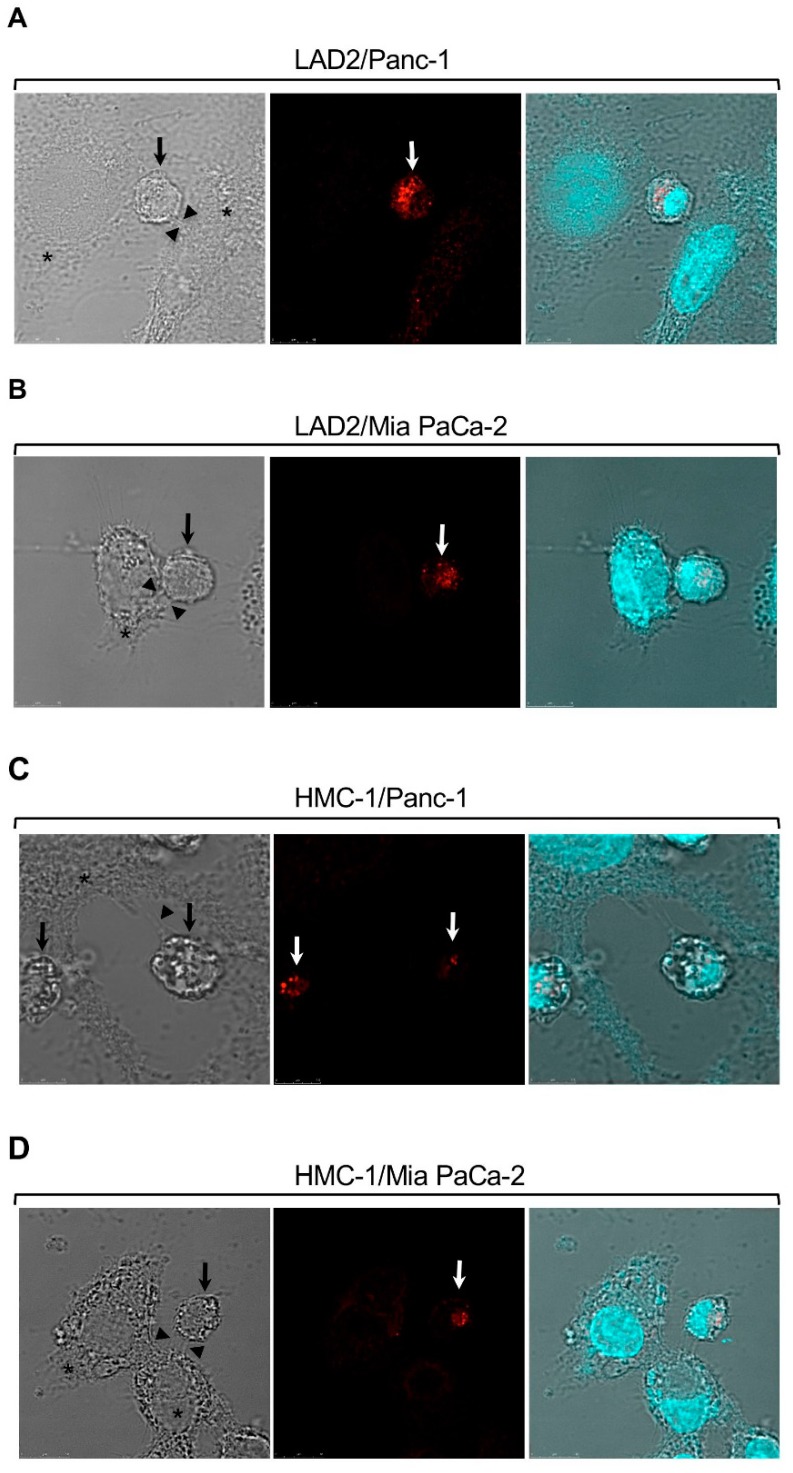
Mast cells (MCs) form synapses with pancreatic cancer cells in co-culture. LAD2 (**A**,**B**) or HMC-1 cells (**C**,**D**) were co-cultured with Panc-1 (**A**,**C**) or Mia PaCa-2 (**B**,**D**) cells at 1:1 ratio. MCs were stained with antibodies directed against tryptase and visualized by confocal microscopy. Bar = 10 μm. Arrows point to MCs, stained in red. Asterisks mark pancreatic cancer cells and arrowheads point to contact areas formed between the MCs and the pancreatic cancer cells. “Reprinted from Cancer Letters, 397, Yaara Gorzalczany, Eyal Akiva, Ofir Klein, Ofer Merimsky and Ronit Sagi-Eisenberg, Mast cells are directly activated by contact with cancer cells by a mechanism involving the autocrine formation of adenosine and autocrine/paracrine signaling of the adenosine A3 receptor. 23-32. Copyright © 2017 with permission from Elsevier”.

**Figure 2:**
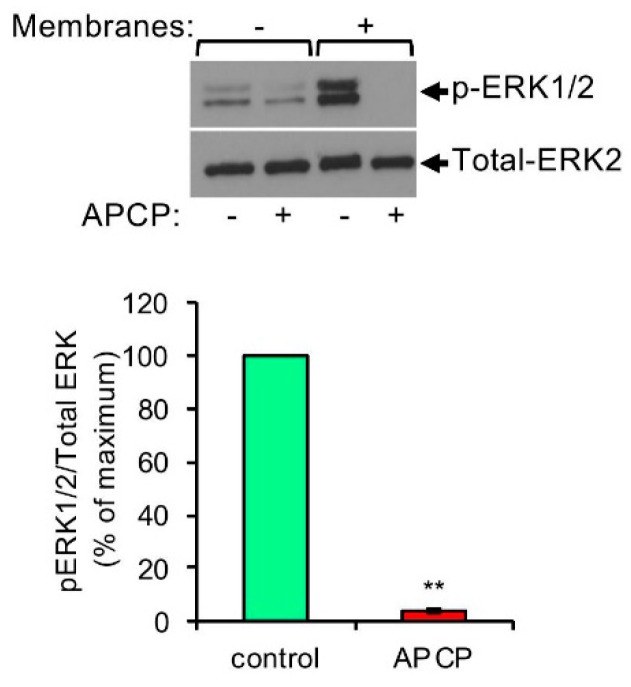
Pancreatic cancer cell derived membranes stimulate ERK1/2 phosphorylation in MCs by a CD73-dependent mechanism. LAD2 cells (1 × 10^6^ cells/mL) were incubated for 30 min in the absence or presence of APCP (5 μM). Cells were then left untreated or treated for 1 min with membranes derived from Mia PaCa-2 pancreatic cancer cells (50 μg/mL), as indicated. Cell lysates were resolved by SDS-PAGE and immunoblotted with anti phospho-ERK1/2 antibodies, followed by reprobing with anti-total-ERK2 as indicated. A representative blot is shown. The intensities of the bands corresponding to phospho-ERK1/2 and total-ERK2 were quantified by densitometry using Image-J software and the relative pixel densities (phosphorylated/total) were calculated. Data are presented as mean ± SEM of three independent experiments. ** *p* = 2.0 × 10^−7^. “Reprinted from Cancer Letters, 397, Yaara Gorzalczany, Eyal Akiva, Ofir Klein, Ofer Merimsky and Ronit Sagi-Eisenberg, Mast cells are directly activated by contact with cancer cells by a mechanism involving the autocrine formation of adenosine and autocrine/paracrine signaling of the adenosine A3 receptor. 23-32, Copyright © 2017 with permission from Elsevier.”

**Figure 3:**
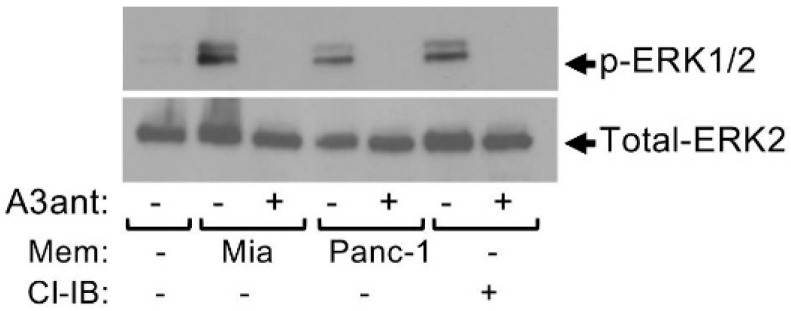
Pancreatic cancer cell derived membranes stimulate ERK1/2 phosphorylation in MCs in an adenosine A3 receptor-dependent manner. LAD2 cells (1 × 10^6^ cells/mL) were incubated for 30 min in the absence or presence of the A3R antagonist MRS1220 (A3ant, 100 nM), as indicated. Cells were then left untreated or treated for 1 min with either membrane (50 μg/mL) derived from Mia PaCa-2 pancreatic cancer cells, or Panc-1 pancreatic cancer cells, or with Cl-IB-MECA (Cl-IB, 100 nM), as indicated. Cell lysates were resolved by SDS-PAGE and immunoblotted with anti phospho-ERK1/2 antibodies, followed by reprobing with anti-total-ERK2 as indicated. A representative blot is shown. “Reprinted from Cancer Letters, 397, Yaara Gorzalczany, Eyal Akiva, Ofir Klein, Ofer Merimsky and Ronit Sagi-Eisenberg, Mast cells are directly activated by contact with cancer cells by a mechanism involving the autocrine formation of adenosine and autocrine/paracrine signaling of the adenosine A3 receptor. 23-32, Copyright © 2017 with permission from Elsevier.”.

Analysis of the crosstalk between MCs and hepatocarcinoma (HCC) using transplantable H22 HCC tumors demonstrated that MCs promote infiltration of myeloid derived suppressor cells (MDSCs) and production of IL-17 by MDSCs [69]. IL-17 then indirectly attracts Treg, enhances their suppressor function and induces their IL-9 production, which in turn, strengthens the pro tumorigenic activity of the MCs in the TME [69]. This vicious cycle involves the upregulation of CD39 and CD73 in Treg, followed by the release of adenosine [69]. These results, which implicate adenosine as pivotal in the crosstalk among the tumor, MCs, MDSCs and Treg, have prompted us to explore the possibility that adenosine may also mediate direct crosstalk between MCs and the tumor. We were particularly intrigued by such a possibility because our previous findings have already recognized autocrine adenosine signaling as involved in the mode of activation of MCs by activated T cells [70]. Hence, we demonstrated that direct activation of MCs by activated T cells, under conditions that recapitulate inflammatory settings, results in the formation of adenosine and autocrine/paracrine signaling by the A3R [70]. In view of our findings that demonstrated that A3R signaling reprograms human MCs by upregulating genes involved in angiogenesis and tissue remodeling [71], which are both tumor favorable processes, we envisioned that MCs might also form synapses with cancer cells leading to their activation. Indeed, imaging of model human mast cell lines (i.e., HMC-1 and LAD2 cells), that were grown in co-culture with either one of two human pancreatic cancer cell lines, Panc-1 or Mia PaCa-2, revealed that the MCs, that are recognized by their positive staining for tryptase, a serine proteinase contained in MC secretory granules and an MC marker, formed contacts with the cancer cells (Figure 1). These results strongly suggested that MCs may indeed be directly activated via contact with cancer cells. To analyze this possibility, we then exposed HMC-1 or LAD2 human MCs, as well as primary mouse bone marrow, derived MCs (BMMCs), to membranes that were isolated from the pancreatic cancer cells, hence, conditions that recapitulate cell contact mediated activation [70,72], and asked if contact with the cancer cell membranes could activate the MCs. Indeed, monitoring phosphorylation of the ERK1/2 MAP kinases, as a reporter for the state of MC activation, demonstrated increased phosphorylation of the MAP kinases (Figure 2), thus documenting for the first-time direct activation of MCs by cell contact with cancer cells. Enhanced phosphorylation of ERK1/2 was dose and time dependent and sensitive to inhibitors of the MEK kinase (i.e., U0126) and phosphatidylinositol 3 kinase (i.e., LY294002) [20], revealing a signaling cascade whereby ERK signaling resides downstream of phosphatidylinositol 3 kinase(s) [20]. Furthermore, consistent with our expectation, we found that cancer cell-triggered activation of the MCs involves the autocrine formation of adenosine. The latter was indicated by the significant, by more than 90%, inhibition of the cancer cell-triggered phosphorylation of ERK1/2 by adenosine 5′-(α, β-methylene) diphosphate (APCP) (Figure 2), a selective inhibitor of CD73, the ecto-nucleotidase that mediates autocrine formation of adenosine [73]. Similarly to activation by T cells, also activation by the pancreatic cancer cells was found to be sensitive to inhibition by MRS1220, a specific antagonist of the A3R (Figure 3). In fact, MRS1220 was as effective in inhibiting phosphorylation of ERK1/2 that was triggered by pancreatic cancer cell derived membranes, as it was in inhibiting ERK1/2 phosphorylation that was induced by Cl-IBMECA, a specific agonist of the A3R (Figure 3). Further studies demonstrated that cancer cell triggered ERK1/2 phosphorylation could also be inhibited by knockdown of the A3R by siRNA or by downregulating the receptor following prolonged exposure to the ligand, i.e., Cl-IBMECA [20].

We have also extended our studies to include two additional cancer types and found that similarly to the pancreatic cancer cell derived membranes, also membranes derived from two non-small cell lung carcinoma cell lines (i.e., A549 and H1299), as well as membranes derived from a leiomyosarcoma cell line (i.e., SK-LMS-1) could activate the MCs and also in both cases, activation was sensitive to inhibition by APCP and MRS1220, demonstrating their dependence on autocrine formation adenosine and signaling by the A3R [20].

**Figure 4:**
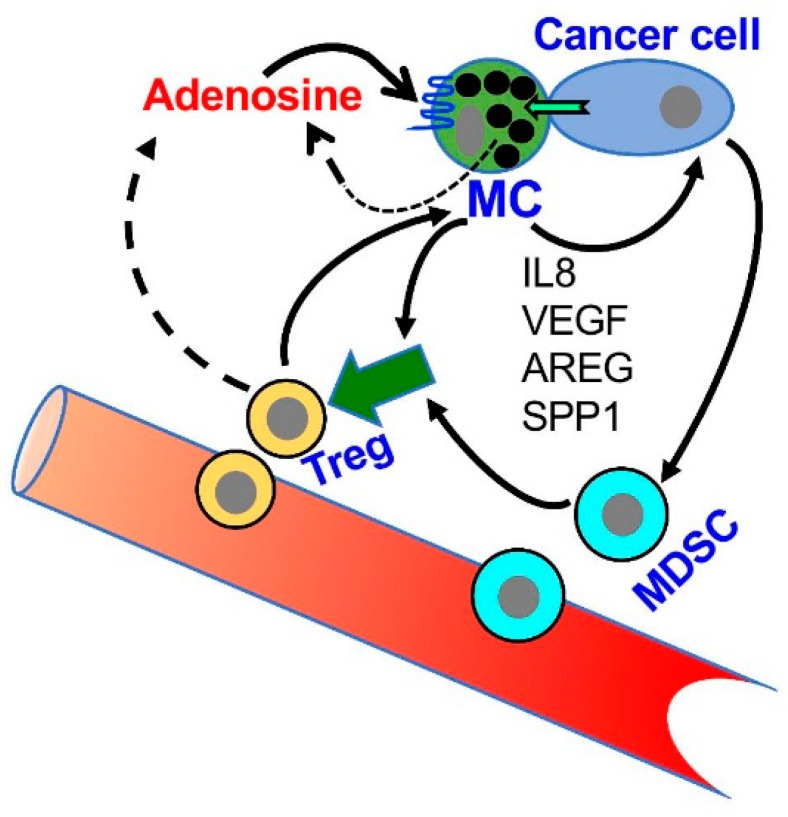
A model of the role of adenosine in the crosstalk among MCs, MDSCs and Treg in the tumor microenvironment (TME). According to this model, MCs migrate to and are activated in the TME; the activated MCs release a panel of factors that influence the attraction and activity of MDSCs and Treg cells (For details see [20,62,64]). In addition, MCs are directly activated by the cancer cells leading to adenosine production and autocrine/paracrine activation of the MCs. Adenosine signaling, that is mediated by the A3R, then leads to the release of angiogenic and tissue remodeling factors, including interleukin 8 (IL8), Vascular endothelial growth factor (VEGF), amphiregulin (AREG) and Secreted Phosphoprotein 1(SPP1, osteopontin) that influence tumor progression.

Therefore, taken together, the results of others and us identify autocrine signaling of adenosine as a central factor in the crosstalk between MCs and tumor cells in the TME. By activating the MCs, autocrine/paracrine signaling of adenosine is likely to mediate the upregulation of angiogenesis and tissue remodeling genes [71], as well as contribute to the immunosuppressive crosstalk with MDSCs and Treg (Figure 4), thus inducing an immunosuppressed TME. Furthermore, this pro-tumorigenic signaling is amplified by the autocrine formation of adenosine by the tumor itself that also expresses the CD39/CD73 ecto-enzymes [46]. It is therefore not surprising that CD39 and CD73 are considered biomarkers of patient outcomes, whose high expression is linked with poorer prognosis [32,52,74,75].

## 7. Future Perspectives

The finding that adenosine is involved in MC-cancer crosstalk, both directly and via complex interactions with other cells of the immune system may explain the controversary concerning the role of MCs in the TME. In view of the fact that adenosine signals through four distinct receptors that differ in their affinity to adenosine, as well as in their functions, it is anticipated that changes in the concentration of adenosine could alter the functional impact of adenosine. For example, while at low concentrations only the high affinity receptors would signal, an increase in adenosine concentration, brought about by the extracellular metabolism of ATP by the CD39/CD73 ecto enzymes, would allow the low affinity adenosine receptors to signal as well. In a similar manner, changes in the relative expression of the receptors may influence the biological outcome. In this regard, it is important to note that we have shown that adenosine itself affects the expression level of the A3R [71]. Therefore, the elevation of adenosine concentrations would affect the overall signaling of adenosine, both by increasing the repertoire of the receptors that signal and by modulating their relative expression levels. Thus, while adenosine signaling is an attractive candidate for drug targeting, caution must be taken, since the outcome of adenosine signaling may change with the tumor type or stage. Another issue that needs to be considered in this regard, is the fact that human adenosine receptors, at least the A3R for that matter, seems to differ in its signaling patterns from that of the rodent receptor [71,76]. This calls for caution when translating preclinical data to clinical data.
